# The Diversity of Hymenoptera on Pummelo (*Citrus maxima* (Burm.) Merr.)

**DOI:** 10.3390/insects17090886

**Published:** 2026-08-24

**Authors:** Caihong Zhang, Xiangzeng Xu, Xiaojiao Zhang, Qinlin Deng, Xue Li, Shide Gao

**Affiliations:** Yunnan Institute of Tropical Crops, Jinghong 666100, China; zchcc1@126.com (C.Z.); xxz351966214@163.com (X.X.); zhangxiaojiaoxwg@163.com (X.Z.); 15938233120@163.com (Q.D.); lixue18783113911@163.com (X.L.)

**Keywords:** pummelo, Hymenoptera, species diversity, community structure

## Abstract

Hymenoptera play important ecological roles in agroecosystems as parasitoids, predators, and pollinators. This study investigated the species composition and diversity of Hymenoptera in a conventionally managed pummelo (*Citrus maxima* (Burm.) Merr.) orchard in Xishuangbanna, Yunnan Province, China, from February 2025 to February 2026. A total of 8446 specimens were collected using Malaise traps and sweep netting, belonging to 11 superfamilies, 37 families, and 191 morphospecies. The community was dominated by parasitoids (140 morphospecies, 73.30%), followed by predators (30 morphospecies, 15.71%), pollinators (18 morphospecies, 9.42%), and phytophagous gall-inducers (3 morphospecies, 1.57%). Ichneumonoidea (42 morphospecies, 21.99%), Chalcidoidea (35 morphospecies, 18.32%), Vespoidea (30 morphospecies, 15.71%) and Apoidea (28 morphospecies, 14.66%) were the dominant superfamilies, collectively accounting for 70.68% of all morphospecies. At the family level, Ichneumonidae (25 morphospecies), Braconidae (17 morphospecies), Bethylidae (13 morphospecies), and Pompilidae and Scelionidae (11 morphospecies each) were the most morphospecies-rich families. Shannon–Wiener diversity was higher at the family level (*H*′ = 2.9340) than at the superfamily level (*H*′ = 2.0302), indicating greater heterogeneity at a finer taxonomic resolution. These results demonstrate that the surveyed pummelo orchard in Xishuangbanna harbors a species-rich and functionally diverse hymenopteran community with substantial potential for biological pest control and pollination services.

## Simple Summary

Wasps and ants help control agricultural pests, while bees pollinate crops. Together, these insects provide essential services that support healthy farming ecosystems. However, little is known about these insects in tropical pummelo orchards in China. This study surveyed these insects in pummelo orchards in Xishuangbanna, Yunnan Province, over one year. A total of 8446 insects were collected, representing 191 morphospecies in 37 families. Most of the species found were tiny parasitic wasps that kill crop pests by laying eggs inside them, making up almost three out of every four species found. Other important groups included hunting wasps that catch pest insects and bees that pollinate flowers. These results show that pummelo orchards support a rich and diverse community of beneficial insects. This knowledge can help farmers adopt practices that protect these helpful insects, such as reducing insecticide use and planting flowers around orchards. The findings also provide a starting point for future research on natural pest control in tropical fruit farming.

## 1. Introduction

Assemblages of Hymenoptera represent a dominant component of arthropod communities in perennial agroecosystems, where they mediate critical ecosystem processes including biological control, pollination, and trophic regulation [1,2]. Parasitoids, in particular, function as core natural enemy resources for biological pest control by ovipositing within or on host arthropods, with larvae completing development through host tissue consumption, thereby suppressing pest populations [3,4]. Predatory groups, including Pompilidae, Formicidae, and Crabronidae, regulate arthropod populations through active hunting, whereas pollinators within the superfamily Apoidea provide essential pollination services for crops and co-flowering plants [5]. Phytophagous gall-inducers such as Cynipidae influence plant–herbivore interactions and food web complexity through gall induction, although some members may also function as inquilines or parasitoids [6]. Thus, comprehensive knowledge of hymenopteran species composition and functional structure in orchard ecosystems is fundamental to understanding agroecosystem service mechanisms and developing integrated pest management strategies.

Citrus is among the most economically important fruit crops globally. China, as the world’s largest producer, accounts for the greatest share of citrus cultivation area and yield [7]. Pummelo (*Citrus maxima* (Burm.) Merr.), also commonly spelled pomelo, is widely cultivated in southern China due to its large fruit size, nutritional value, and high economic returns. This study focused on pummelo orchards in Jinghong City, Xishuangbanna Dai Autonomous Prefecture, Yunnan Province, a tropical region renowned for its exceptionally high biodiversity. However, the expansion and intensification of pummelo production have been accompanied by increasing pest pressure, while excessive reliance on chemical pesticides has disrupted orchard ecosystem balance and depleted natural enemy resources, posing severe challenges to sustainable production [8,9].

Previous studies on insect communities in Chinese pummelo orchards have yielded valuable survey data, albeit with important limitations. Insect communities in Meizhou, Guangdong Province, were surveyed, identifying Ichneumonidae as the dominant natural enemy family and Noctuidae as the principal pest family, with diversity indices exhibiting marked seasonal fluctuations [10]. Twenty-five insect species (7 orders, 20 families) were documented in a honey pummelo orchard in Zhangzhou, Fujian Province, where natural enemy insects accounted for merely 29.41% of total species richness [11]. Inter-row ground cover management significantly influenced insect community structure in Sizhou pummelo orchards, Guizhou Province, with *Paspalum notatum* and *Lolium perenne* treatments showing higher beneficial-to-pest ratios [12]. Notably, these investigations were conducted in subtropical regions and targeted either general insect fauna or pest assemblages, lacking systematic surveys specifically focused on Hymenoptera.

Research on hymenopteran diversity in tropical pummelo orchards remains particularly scarce. A survey of arthropods in lemon orchards in Dehong, Yunnan Province, was conducted, but hymenopterans were reported only at the ordinal level, precluding lower-taxon identification and analysis [13]. Regarding methodological advances, DNA metabarcoding was employed to assess insect diversity in citrus orchards in Ganzhou, Jiangxi Province, recovering 2141 Barcode Index Numbers (BINs) from pooled Malaise trap samples [14]. While this study demonstrated the potential of molecular approaches for orchard biodiversity assessment, comparison against the BOLD database revealed limited barcode reference sequence coverage for Chinese citrus insects, constraining the widespread application of metabarcoding in the region.

In summary, previous studies exhibit three principal limitations: (1) a geographic bias toward subtropical regions, with insufficient attention to tropical pummelo orchards; (2) a taxonomic focus on general insect assemblages or pest complexes, rather than systematic investigations of Hymenoptera; and (3) limited use of molecular identification tools in orchard Hymenopteran surveys. To address these gaps, the present study systematically sampled hymenopteran communities in pummelo orchards in Jinghong City, Xishuangbanna, a tropical region in southwestern China, using Malaise traps and sweep netting and combined morphological identification with COI DNA barcoding. The specific objectives were to characterize the species composition and community structure of Hymenoptera at multiple taxonomic levels, to quantify community diversity using multiple indices and assess scale-dependent diversity patterns, and to classify taxa into functional guilds (parasitoids, predators, pollinators, and phytophagous gall-inducers) to evaluate their potential for providing ecosystem services in pummelo agroecosystems.

## 2. Materials and Methods

### 2.1. Overview of the Study Area

This study was conducted from February 2025 to February 2026 in a pummelo orchard located in Jinghong City, Xishuangbanna Dai Autonomous Prefecture, Yunnan Province, China (21°27′–22°36′ N, 100°25′–101°31′ E). The region is characterized by a tropical monsoon climate, with mean annual temperatures ranging from 18.6 to 22.9 °C, annual precipitation of 1140–1700 mm, and altitudes ranging from 485 to 2196.8 m above sea level. The pummelo variety employed in this study was the native *Citrus maxima* ‘Dongshizao’, indigenous to Xishuangbanna. The sampled orchard consisted of 6.67 hectares (100 mu) of pummelo trees aged 6–26 years, maintained as a standardized demonstration base under conventional cultivation with two pesticide applications annually (in June and September). All pesticides used were biological pesticides; applications were restricted to these two months, avoiding the peak hymenopteran activity periods of March–May and October–November.

### 2.2. Sampling Methods

Two systematic sampling methods were employed: (1) Malaise trapping: Five Malaise traps (Townes-style, with black netting and white roofs) were positioned across the pummelo-growing area in a zigzag pattern, with inter-trap distances of approximately 80–100 m to minimize spatial autocorrelation while covering the 6.67 ha area. Traps were placed at the edge of tree rows, approximately 1.5 m inside the canopy border, with collection bottles containing 75% ethanol (prepared from absolute ethanol; Tianjin ZhiYuan Reagent Co., Ltd., Tianjin, China). Traps were operated continuously, and specimens were collected monthly (approximately every 30 days) throughout the study period. Each trapping interval lasted 28–31 days, yielding 12 collection events per trap. (2) Sweep netting: Sweep netting was conducted every two weeks (24 sampling events total) along five predetermined transects (each 100 m long) that bisected the orchard diagonally. Along each transect, three replicate sweep samples were performed using a standard insect sweep net (38 cm diameter, 60 mesh), with each replicate consisting of 50 consecutive sweeps through the herbaceous understory and lower canopy foliage (up to 2 m height). The total sweep effort was therefore 5 transects × 3 replicates × 50 sweeps = 750 sweeps per sampling event or 18,000 sweeps over the study period. All sampling events were conducted between 09:00 and 11:30 h on days without rain to standardize weather conditions [15].

### 2.3. Specimen Identification and Preservation

All collected specimens were fixed and stored in 75% ethanol and examined under a stereomicroscope (Olympus SZX7, Olympus Corporation, Tokyo, Japan) for morphological identification. Preliminary taxonomic assignments were made by reference to the pertinent taxonomic literature and identification keys [16,17,18,19,20,21,22,23]. Identifications were verified by two independent researchers; discrepancies were resolved by microscopic re-examination of diagnostic characters. The higher-level classification (superfamily and family assignments) follows Goulet et al. and Aguiar et al. [19,24], as implemented in the Hymenoptera Name Server (https://www.waspweb.org/, accessed on 15 July 2026). Family-level placements were cross-referenced with recent phylogenomic studies where applicable [25,26]. This framework was applied consistently to all identified taxa, and the resulting superfamily and family totals were rechecked against the adopted system to ensure internal consistency. Functional guild assignments (parasitoids, predators, pollinators, and phytophagous gall-inducers) were based on family-level feeding habits and ecological functions documented in the literature [27,28,29,30,31].

For specimens of uncertain identity, genomic DNA was extracted and the mitochondrial cytochrome *c* oxidase subunit I (COI) barcode region was amplified and sequenced to assist in species confirmation [32,33]. The mitochondrial cytochrome c oxidase subunit I (COI) barcode region was amplified using the primers BF3 (5′-CCHGAYATRGCHTTYCCHCG-3′) and BR2(5′-TCDG GRTGNCCRAARAAYCA-3′) [32] in 25 µL reactions. Bidirectional Sanger sequencing was performed by Aiji Biotechnology Co., Ltd. (Guangzhou, China), on an ABI 3730 platform. Raw sequences were assembled, trimmed, and edited in MEGA v.11. Edited sequences were queried against the NCBI GenBank database using BLASTn (BLAST+ 2.13.0, NCBI, Bethesda, MD, USA). Species assignments were based on ≥98% sequence similarity to reference vouchers, supplemented by morphological congruence. For specimens lacking close reference matches (<98%), sequences were submitted to GenBank and treated as morphologically identified species with molecular confirmation pending.

Voucher specimens are preserved in Laboratory 329, Citrus Research Group, Yunnan Institute of Tropical Crops, Jinghong, Yunnan Province, China.

### 2.4. Data Analysis Methods

The following diversity indices were calculated:

Shannon–Wiener diversity index (*H*′) [34]:

*H*′ = −Σ *p_i_* ln *p_i_*, (1)

where *p_i_* = *N_i_*/*N*, *N_i_* is the number of individuals in taxonomic unit *i*, and *N* is the total number of individuals.

Simpson’s diversity index (*D*) [35]:

*D* = 1 − Σ(*p_i_*)^2^, (2)

where *p_i_* is the proportion of individuals in taxonomic unit *i*. *D* represents the probability that two randomly selected individuals belong to different taxonomic units.

Pielou’s evenness index (*J*) [36]:

*J* = *H*′/ln *S*, (3)

where *H*′ is the Shannon–Wiener diversity index and *S* is the total number of taxonomic units. *J* represents the degree of evenness in the distribution of individuals among taxonomic units.

Margalef richness index (*R*) [37]:

*R* = (*S* − 1)/ln *N*, (4)

where *S* is the total number of taxonomic units and *N* is the total number of individuals.

Diversity analyses were performed using the “vegan” package (version 2.6-2) [38] in R software (version 4.2.0) [39]. Graphs and figures were prepared using Origin 2021 (OriginLab Corporation, Northampton, MA, USA).

## 3. Results

### 3.1. Morphospecies Composition

A total of 8446 hymenopteran specimens were collected in this study, belonging to 11 superfamilies, 37 families, and 191 morphospecies. COI barcoding was applied to 24 specimens representing 16 morphospecies across 8 families to assist in species-level confirmation. The complete inventory of all 191 morphospecies-level units, including voucher specimen IDs and GenBank accession numbers where applicable, is provided in Supplementary Table S3. Of the 191 morphospecies, 14 were assigned formal species names based on detailed morphological examination and comparison with voucher material; a subset were further supported by COI barcode sequencing (GenBank accessions PZ761219–PZ761242). The remainder are retained as tentative morphospecies. All community-level analyses are aggregated at the family and superfamily levels and remain robust regardless of species-level nomenclatural status. Species richness was highest in Ichneumonoidea (42 morphospecies, 21.99%), followed by Chalcidoidea (35 morphospecies, 18.32%), Vespoidea (30 morphospecies, 15.71%), and Apoidea (28 morphospecies, 14.66%). These four superfamilies collectively comprised 135 morphospecies (70.68%). Chrysidoidea (16 morphospecies, 8.38%), Cynipoidea (12 morphospecies, 6.28%), and Platygastroidea (11 morphospecies, 5.76%) represented intermediate contributors, whereas Proctotrupoidea (9 morphospecies, 4.71%), Evanioidea (5 morphospecies, 2.62%), Ceraphronoidea (2 morphospecies, 1.05%), and Mymarommatoidea (1 morphospecies, 0.52%) were comparatively minor components (Table 1).

At the family level, morphospecies richness was highest in Ichneumonidae (25 morphospecies, 13.09%), followed by Braconidae (17 morphospecies, 8.90%), Bethylidae (13 morphospecies, 6.81%), Scelionidae (11 morphospecies, 5.76%), and Pompilidae (11 morphospecies, 5.76%), which collectively comprised 77 morphospecies (40.31%). Halictidae (9 morphospecies, 4.71%), Formicidae (9 morphospecies, 4.71%), Eulophidae (9 morphospecies, 4.71%), Apidae (7 morphospecies, 3.66%), and Diapriidae (7 morphospecies, 3.66%) represented substantial contributors, whereas the remaining 27 families, each containing six or fewer species, accounted for 73 morphospecies (38.22%) and were comparatively minor components individually (Table 2). The distribution of specimen abundance across families by collection method is provided in Supplementary Table S4.

### 3.2. Community Structure Characteristics

#### 3.2.1. Superfamily-Level Community Structure

**Figure 1:**
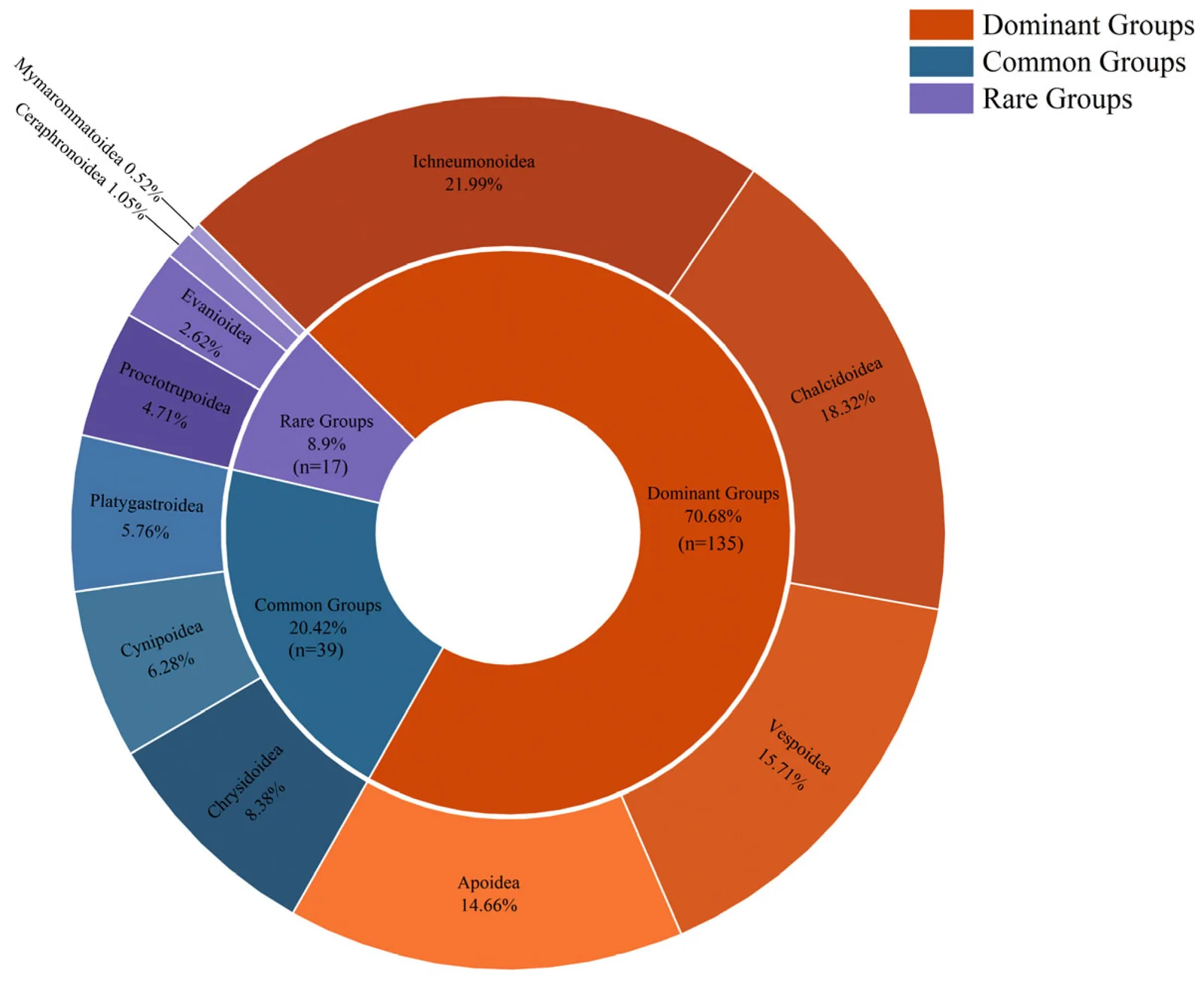
Classification of 11 hymenopteran superfamilies by relative morphospecies richness in pummelo orchards, Xishuangbanna, Yunnan Province, China (*N* = 191 total morphospecies). Inner ring: dominant (≥10%, *n* = 135), common (5–10%, *n* = 39), and rare (<5%, *n* = 17) groups. Outer ring: proportional contribution of each superfamily.

Based on the relative morphospecies richness of each superfamily, the Hymenoptera community in pummelo orchards was classified into three groups: dominant (≥10%), common (5–10%), and rare (<5%) (Figure 1).

The dominant group comprised four superfamilies—Ichneumonoidea, Chalcidoidea, Vespoidea, and Apoidea, which collectively accounted for 135 morphospecies (70.68% of the total). Among these, Ichneumonoidea was the most morphospecies-rich, contributing 42 morphospecies (21.99%) and representing more than one-fifth of the recorded community. The common group encompassed Chrysidoidea, Cynipoidea, and Platygastroidea, totaling 39 morphospecies (20.42%), while the rare group comprised Proctotrupoidea, Evanioidea, Ceraphronoidea, and Mymarommatoidea, together representing 17 morphospecies (8.9%).

#### 3.2.2. Family-Level Community Structure

**Figure 2:**
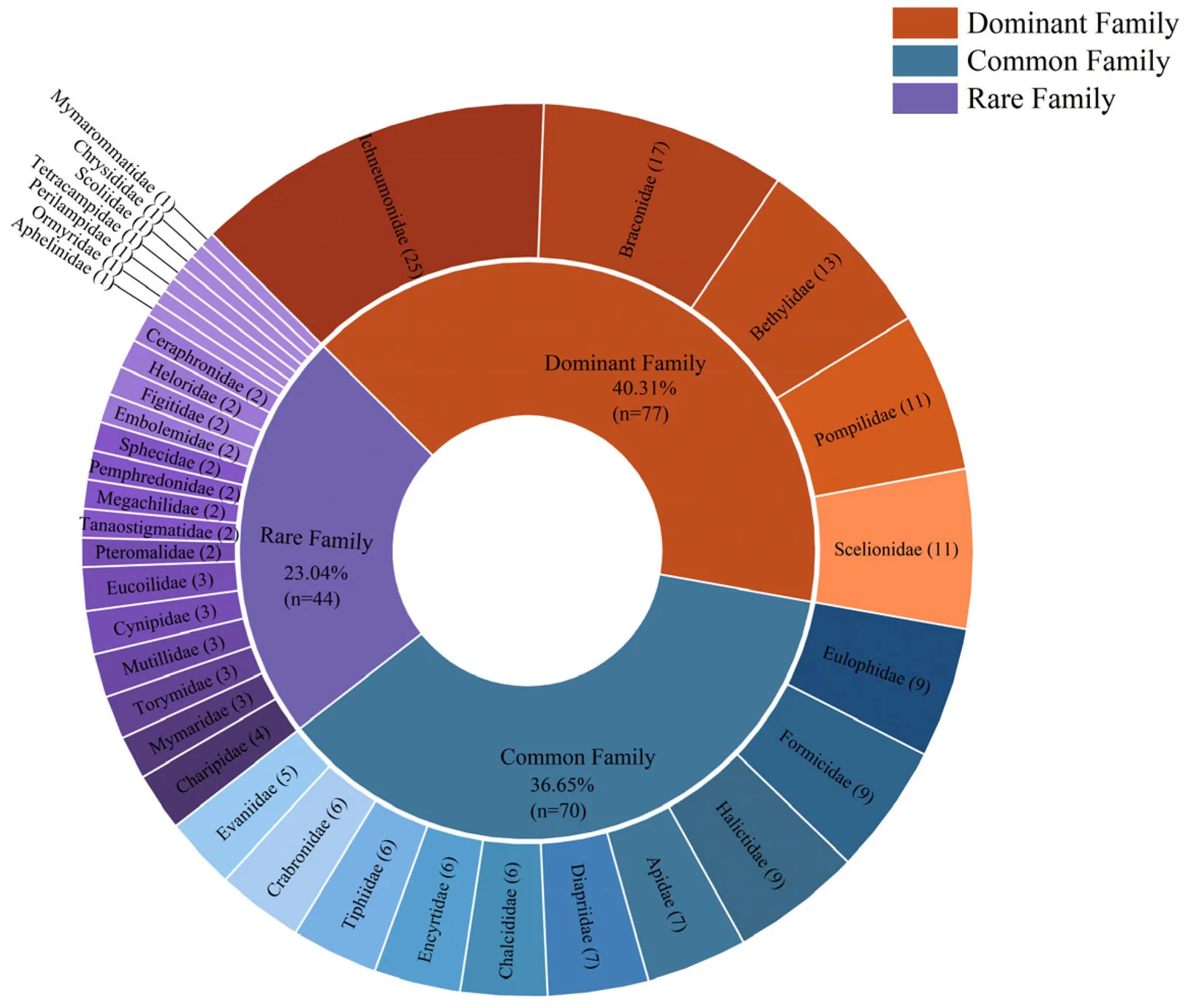
Classification of 37 hymenopteran families by morphospecies richness in pummelo orchards, Xishuangbanna, Yunnan Province, China (*N* = 191 total morphospecies). Families are grouped into three dominance categories: dominant (≥10 morphospecies, *n* = 77), common (5–9 morphospecies, *n* = 70), and rare (≤4 morphospecies, *n* = 44). Outer ring: proportional contribution of each family.

Based on absolute morphospecies counts per family, the 37 hymenopteran families were classified into three categories: dominant (≥10 morphospecies), common (5–9 morphospecies), and rare (≤4 morphospecies) (Figure 2). We employed absolute thresholds at the family level rather than proportional cut-offs (as used for superfamilies) because the 37 families exhibited highly heterogeneous morphospecies richness; proportional thresholds would classify the majority (22 of 37, each contributing <2%) as rare, obscuring meaningful distinctions among moderately diverse families. Absolute species-count categories are standard in family-level arthropod community analyses [10,40].

Five families were classified as dominant: Ichneumonidae (25 morphospecies, 13.09%), Braconidae (17 morphospecies, 8.90%), Bethylidae (13 morphospecies, 6.81%), Scelionidae (11 morphospecies, 5.76%), and Pompilidae (11 morphospecies, 5.76%), which together accounted for 77 morphospecies (40.31% of total morphospecies). Ichneumonidae was the most morphospecies-rich family, followed by Braconidae. Ten families fell into the common category: Halictidae, Formicidae, Eulophidae (9 morphospecies each), Apidae, Diapriidae (7 morphospecies each), Chalcididae, Encyrtidae, Tiphiidae, Crabronidae (6 morphospecies each), and Evaniidae (5 morphospecies), totaling 70 morphospecies (36.65%). The remaining 22 families were classified as rare, accounting for 44 morphospecies (23.04%). These included one family with four species, five families with three species each, eight families with two species each, and eight monotypic families.

#### 3.2.3. Functional Composition

**Figure 3:**
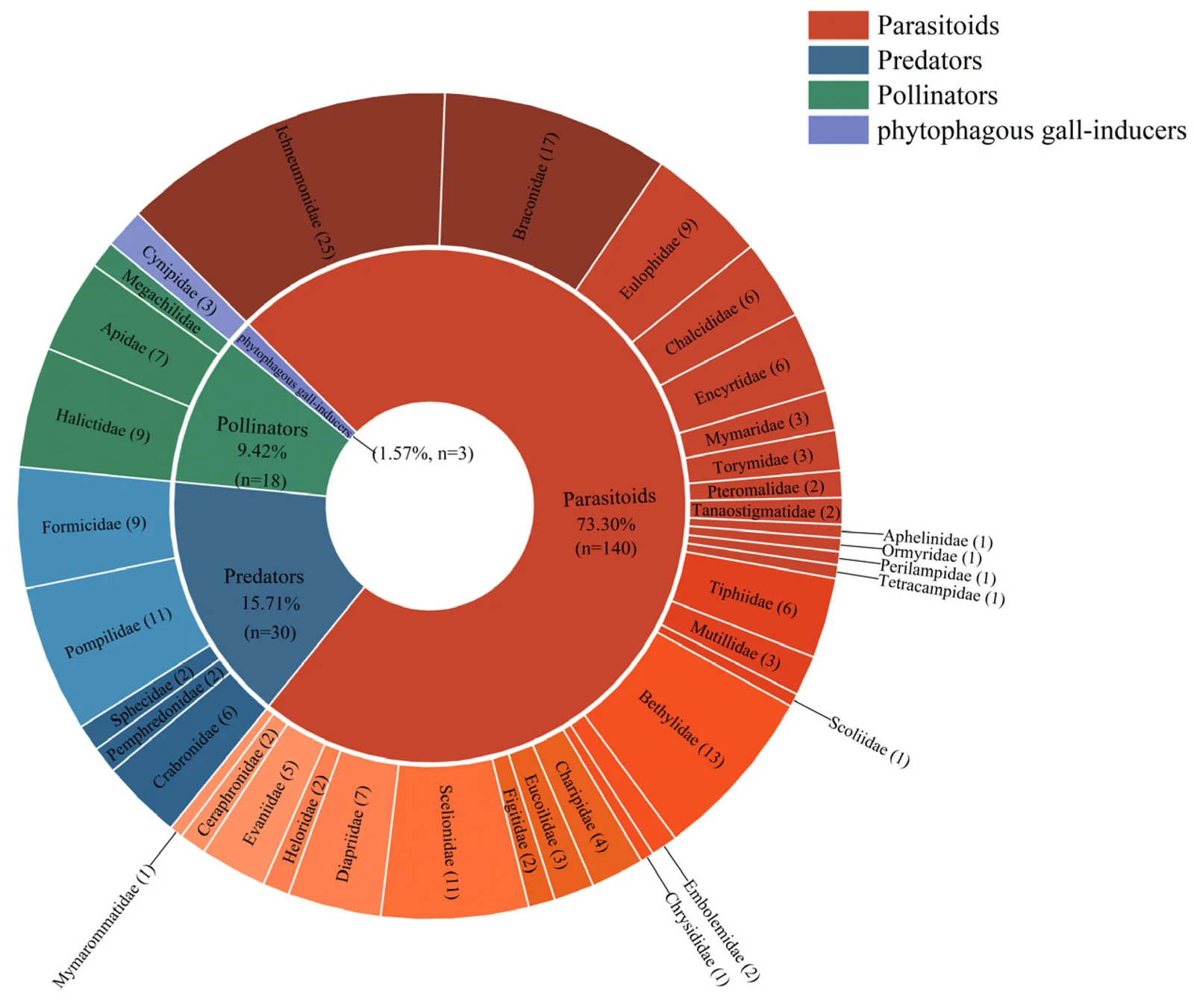
Classification of hymenopteran morphospecies into four functional guilds in pummelo orchards, Xishuangbanna, Yunnan Province, China (*N* = 191 total morphospecies). Morphospecies were categorized by feeding habits and ecological functions into four guilds: parasitoids (*n* = 140), predators (*n* = 30), pollinators (*n* = 18), and phytophagous gall-inducers (*n* = 3). Note: Guild assignments reflect family-level ecological generalizations; individual morphospecies may deviate from these generalized roles.

According to their family-level feeding habits and ecological functions, the hymenopteran morphospecies were categorized into four functional guilds: parasitoids, predators, pollinators and phytophagous gall-inducers (Figure 3).

Parasitoids constituted the dominant functional group, encompassing 140 morphospecies from 24 families and accounting for 73.30% of all recorded morphospecies. This guild exhibited substantial phylogenetic diversity, spanning eight superfamilies (Ichneumonoidea, Chalcidoidea, Chrysidoidea, Platygastroidea, Proctotrupoidea, Evanioidea, Ceraphronoidea, and Mymarommatoidea), as well as the parasitoid families within Vespoidea (Tiphiidae, Mutillidae, and Scoliidae) and Cynipoidea (Charipidae, Eucoilidae, and Figitidae). Predators encompassed 30 morphospecies distributed across five families, accounting for 15.71% of the total morphospecies richness. This functional group was represented by Pompilidae (11 morphospecies), Formicidae (9 morphospecies), Crabronidae (6 morphospecies), Pemphredonidae (2 morphospecies), and Sphecidae (2 morphospecies). Pollinators comprised 18 morphospecies from three families, accounting for 9.42% of the total morphospecies recorded. Halictidae (9 morphospecies) was the most species-rich pollinator family, followed by Apidae (7 morphospecies) and Megachilidae (2 morphospecies). Phytophagous gall-inducers comprised solely Cynipidae (3 morphospecies, 1.57%).

In summary, the functional composition of the hymenopteran community was dominated by parasitoids (73.30%), with predators (15.71%) and pollinators (9.42%) as secondary components, and phytophagous gall-inducers representing only 1.57% of the total.

### 3.3. Diversity Indices

#### 3.3.1. Superfamily-Level Diversity

At the superfamily level, the Shannon–Wiener diversity index (*H*′) was 2.0302, the Simpson’s diversity index (*D*) was 0.8498, the Pielou’s evenness index (*J*) was 0.8466, and the Margalef richness index (*R*) was 1.1060.

#### 3.3.2. Family-Level Diversity

At the family level, the Shannon–Wiener diversity index (*H*′) was 2.9340, the Simpson’s diversity index (*D*) was 0.9241, the Pielou’s evenness index (*J*) was 0.8125, and the Margalef richness index (*R*) was 3.9817.

## 4. Discussions

### 4.1. Overview of Hymenopterans in Pummelo Orchards

The present study documents the hymenopteran community associated with a single pummelo orchard in Xishuangbanna, southwestern China, based on the collection of 8446 specimens belonging to 11 superfamilies, 37 families, and 191 morphospecies. To our knowledge, this represents the first morphospecies-level inventory of Hymenoptera in tropical pummelo agroecosystems in China. Previous surveys of pummelo-associated insects in subtropical China [11,40] and tropical Indonesia [41] reported hymenopterans at broader taxonomic levels (family or order) without enumerating morphospecies, precluding direct quantitative comparisons of morphospecies richness. The 191 morphospecies recorded here provide the first quantitative reference for hymenopteran diversity in tropical pummelo orchards in China, filling a geographic gap between subtropical citrus systems and broader Southeast Asian tropical agroecosystems. It should be noted that the 191 morphospecies-level units include confirmed binomials and tentative morphospecies within microhymenoptera (Supplementary Table S3); the family- and superfamily-level community patterns reported here, however, remain robust to this nomenclatural uncertainty.

While spatial replication was limited to one site, this orchard was deliberately selected following extensive preliminary reconnaissance. Surveys of surrounding commercial pummelo orchards in Xishuangbanna indicated that routine management typically involves high-frequency pesticide applications, often monthly or more during the growing season, which have severely suppressed local arthropod diversity. In contrast, the sampled orchard functions as a standardized demonstration base maintained under conventional cultivation with only 1–2 pesticide applications annually. This reduced-chemical regime provided a unique opportunity to document the hymenopteran community in a tropical pummelo agroecosystem where natural enemy populations have not been eradicated by intensive spraying. Consequently, the findings should be interpreted as a quantitative baseline for this specific, low-spray management regime rather than as representative of all pummelo production systems in the region.

Xishuangbanna’s tropical monsoon climate, characterized by high annual rainfall (1140–1700 mm) and warm temperatures (18.6–22.9 °C) [42], provides conditions conducive to year-round hymenopteran activity, which likely contributes to the high morphospecies richness documented here. However, because climatic variables and host availability were not experimentally manipulated or directly measured in this study, this interpretation remains a plausible hypothesis rather than a demonstrated causal relationship. The assemblage was strongly skewed toward parasitoids (140 morphospecies, 73.30%), with predators, pollinators, and phytophagous gall-inducers representing minor components. Future multi-site studies spanning gradients of pesticide use intensity are needed to assess how management regimes shape hymenopteran diversity across tropical citrus landscapes.

### 4.2. Superfamily-Level Community Structure and Morphospecies Dominance

Categorization of the 11 superfamilies by relative morphospecies richness into dominant (≥10%), common (≥5% to <10%), and rare (<5%) groups revealed a strongly right-skewed distribution. Four superfamilies, Ichneumonoidea, Chalcidoidea, Vespoidea, and Apoidea, collectively accounted for 70.68% of all recorded morphospecies, constituting the dominant assemblage. This pattern is consistent with the prevalence of dominant-morphospecies structures frequently reported in diverse tropical insect assemblages [43,44]. The co-dominance of Ichneumonoidea and Chalcidoidea (40.31% combined) reflects the high representation of these two morphospecies-rich parasitoid lineages in the study system. Their prevalence in pummelo orchards is attributable to the availability of diverse herbivorous hosts, particularly lepidopteran larvae and hemipteran sap-suckers, associated with citrus agroecosystems.

The three common superfamilies, Chrysidoidea (8.38%), Cynipoidea (6.28%), and Platygastroidea (5.76%), contributed 20.42% of total morphospecies richness, functioning as subdominant but ecologically significant components. Platygastroidea, in particular, has been recorded as a specialized parasitoid of hemipteran eggs, a trait with ecological relevance for controlling sap-sucking pests in citrus [1]. Four rare superfamilies, Proctotrupoidea (4.71%), Evanioidea (2.62%), Ceraphronoidea (1.05%), and Mymarommatoidea (0.52%), collectively comprised 8.90% of morphospecies. Their low morphospecies richness may reflect narrow host ranges, specialized microhabitat requirements, or lower detectability by the sampling methods employed. Ampulicidae [45], for example, is a specialized parasitoid of cockroach oothecae, a scarce niche resource in managed orchard environments.

The multi-dominant structure of the hymenopteran community, characterized by the absence of a single overwhelmingly dominant superfamily and the presence of multiple co-dominant lineages, indicates functional redundancy among major taxonomic lineages. This redundancy likely enhances community resilience to environmental perturbations and management disturbances, thereby supporting ecosystem stability in pummelo orchards.

It should also be noted that the observed superfamily-level structure reflects in part the complementary nature of the two sampling methods employed. Malaise traps and sweep netting captured distinct community subsets (Supplementary Table S4): the former were more effective for flying parasitoids (Ichneumonoidea and Chalcidoidea), whereas the latter yielded more ground-active and vegetation-dwelling taxa (Formicidae and Crabronidae). This methodological complementarity supports the combined use of both techniques for comprehensive hymenopteran inventories in orchard ecosystems.

### 4.3. Family-Level Composition and Dominant Families

At the family level, the community was structured around five core families—Ichneumonidae, Braconidae, Bethylidae, Pompilidae, and Scelionidae—which collectively comprised 40.32% of the total morphospecies pool. Ichneumonidae (25 morphospecies, 13.09%) and Braconidae (17 morphospecies, 8.90%) were the most morphospecies-rich families, their co-dominance reflecting the central role of these two largest parasitoid lineages in regulating herbivore populations within pummelo orchards. Bethylidae (13 morphospecies, 6.81%), Scelionidae (11 morphospecies, 5.76%), and Pompilidae (11 morphospecies, 5.76%) constituted the remaining core families, contributing to parasitoid and predator diversity, respectively. The dominance of Ichneumonidae in our study corroborates its prevalence as a natural enemy taxon in subtropical Chinese pummelo systems [10]. Ichneumonid and braconid wasps attack a broad spectrum of hosts, including lepidopteran larvae and coleopteran pests that are major consumers of citrus foliage and fruit [1], thereby providing natural pest suppression services.

Chalcidoidea exhibited the highest family-level diversity, encompassing 11 families and 35 morphospecies (18.32%). Within this superfamily, Eulophidae (9 morphospecies, 4.71%), Chalcididae (6 morphospecies, 3.14%), and Encyrtidae (6 morphospecies, 3.14%) were the dominant families, all of which include species with biocontrol potential against whiteflies, aphids, and scale insects, economically important pests of citrus worldwide [1]. The diversity of Chalcidoidea indicates complementary host-use strategies, with Eulophidae, Chalcididae, and Encyrtidae targeting sap-sucking pests, while other families exploit concealed herbivores, collectively broadening the biocontrol niche space.

Bethylidae (13 morphospecies, 6.81%) and Scelionidae (11 morphospecies, 5.76%) were significant components of the parasitoid fauna. Bethylidae are idiobiont ectoparasitoids of concealed larvae, primarily Coleoptera and Lepidoptera, while Scelionidae are specialized egg parasitoids of Orthoptera and Hemiptera [46,47,48]. The functional complementarity among these parasitoid families, manifested through attack on different host life stages and occupation of distinct microhabitats, contributes to the overall biocontrol potential of the hymenopteran community.

### 4.4. Functional Guilds and Ecosystem Service Potential

The hymenopteran community was classified into four functional guilds (parasitoids, predators, pollinators, and phytophagous gall-inducers), revealing a community strongly skewed toward parasitoid-mediated ecosystem services. Parasitoids accounted for 73.30% of hymenopteran morphospecies richness, a proportion substantially higher than the ranges generally reported for temperate agroecosystems. This predominance is attributable to the year-round growing conditions in Xishuangbanna, which sustain continuous herbivore populations and consequently stable parasitoid communities. Spanning ten superfamilies, the parasitoid assemblage reflects the convergence of multiple independent evolutionary lineages on the parasitoid life history strategy, a pattern consistent with the high diversification rates of parasitoid Hymenoptera in tropical regions [49]. Parasitoids include both endoparasitic and ectoparasitic species that exert top-down regulation of pest populations in pummelo orchards. Families such as Eulophidae, Chalcididae, and Encyrtidae include species with biocontrol potential against sap-sucking pests (whiteflies, aphids, and scale insects), suggesting their applicability in integrated pest management programs.

Predators accounted for 15.71% of morphospecies richness and were dominated by Pompilidae (11 morphospecies, 5.76%), Formicidae (9 morphospecies, 4.71%), and Crabronidae (6 morphospecies, 3.14%). Formicidae were tentatively classified as predators for the purpose of this survey, based on the predominantly predatory ecology of the genera collected (*Oecophylla*, *Pheidole*, etc.), which are frequently documented as arthropod hunters in tropical agroecosystems [50]. It is acknowledged that Formicidae as a family encompasses diverse feeding strategies, including omnivory and hemipteran-tending; however, the species assemblage encountered here is dominated by taxa whose primary functional contribution is predation. Pompilidae, as spider-hunting wasps, occupy a unique predatory niche distinct from other hymenopteran predators, contributing to spider population regulation in the orchard understory. Crabronidae, predators of small insects including dipterans, aphids, and leafhoppers, occupy a complementary functional niche. The co-occurrence of diverse predatory lineages, together with the rich parasitoid community, indicates a multi-layered natural enemy system that provides complementary pest suppression in pummelo orchards.

Pollinators, represented exclusively by three families within Apoidea (Halictidae, Apidae, and Megachilidae), contributed 9.42% of morphospecies richness. Although modest in proportional representation, this guild provides essential pollination services to pummelo and co-flowering plants within and adjacent to orchards. The specialized pilose body structures of adult bees facilitate pollen collection and adherence during foraging, enhancing their efficiency as pollen vectors. Pummelo varieties exhibit self-incompatibility, rendering insect pollinators indispensable for fruit set and yield [5]. The presence of Halictidae (9 morphospecies) and Apidae (7 morphospecies) in the present study indicates that analogous pollination services operate in Xishuangbanna pummelo orchards. Notably, the pollinator guild was confined to three families within Apoidea, indicating a narrow phylogenetic basis for pollination services in this agroecosystem.

The phytophagous gall-inducers guild was represented exclusively by Cynipidae (3 morphospecies, 1.57%). As members of this family neither prey upon other arthropods nor participate in plant pollination, their ecological role in the pummelo orchard community is primarily expressed through plant–herbivore interactions. Cynipidae are predominantly phytophagous, with females inducing gall formation via oviposition-triggered abnormal proliferation of plant tissues in which larvae complete their development. Although morphospecies richness was low in this study, gall wasps exert localized effects on host plant physiology and serve as resources for parasitoid lineages (Figitidae, endoparasitoids of cynipid larvae), thereby contributing indirectly to food web complexity. The low representation of phytophagous Hymenoptera indicates that plant-feeding wasps constitute a minor component of the pummelo orchard community, likely because managed citrus systems are dominated by non-hymenopteran herbivores such as psyllids, scale insects, and mites [1,10,40].

In summary, the hymenopteran community in the pummelo orchards was functionally dominated by parasitoids, which accounted for 73.30% of total morphospecies richness, followed by predators (15.71%) and pollinators (9.42%), whereas phytophagous gall-inducers comprised a negligible proportion (1.57%). The predominance of parasitoids indicates an abundant natural enemy resource base in this tropical agroecosystem, contributing to pest population suppression. The co-occurrence of diverse parasitoid, predator, and pollinator guilds indicates the capacity of this community to provide multiple ecosystem services, namely biological pest control and pollination. The functional structure of this community, dominated by parasitoids with complementary predator and pollinator components, suggests that pummelo orchards can simultaneously support biological pest control and pollination, provided that management practices preserve guild-specific habitat requirements.

It is important to note that the present study documents morphospecies composition and richness patterns; direct measurement of ecosystem processes (e.g., parasitism rates, predation events, flower visitation rates, or pollination effectiveness) was beyond the scope of this survey. Therefore, the functional roles discussed here represent potential ecosystem services inferred from family-level ecological traits and documented host associations in the literature, rather than functions directly demonstrated by the present study.

### 4.5. Influence of Taxonomic Resolution on Diversity Indices

Comparative analysis of diversity indices at the superfamily and family levels reveals how taxonomic resolution affects calculated values. At the family level, the Shannon–Wiener index (*H*′ = 2.9340) and Simpson’s diversity index (*D* = 0.9241) were substantially higher than their superfamily-level estimates (*H*′ = 2.0302; *D* = 0.8498). These differences are expected because the same 8446 specimens were partitioned into 37 families rather than 11 superfamilies, and they reflect the mathematical sensitivity of these indices to the number of taxonomic units rather than an independent ecological pattern. Consequently, this comparison serves primarily as a methodological caution: when reporting diversity indices for higher-level taxa, the taxonomic resolution must be explicitly stated, and values derived at different resolutions should not be directly compared across studies.

Pielou’s evenness index was slightly lower at the family level (*J* = 0.8125) than at the superfamily level (*J* = 0.8466). This difference arises from the mathematical structure of the index (*J* = *H*′/ln *S*): although *H*′ increased by 44.5% from the superfamily to the family level, the denominator ln(*S*) increased by 50.5% (from ln(11) = 2.398 to ln(37) = 3.611), resulting in a marginally lower *J* value. The modest difference in *J* between the two scales (Δ*J* = 0.034) reflects that the proportional increase in H′ was offset by the proportional increase in ln(S), leaving the evenness estimate relatively stable across resolutions.

The most pronounced sensitivity was observed for Margalef richness (*R* = 3.9817 at the family level vs. *R* = 1.1060 at the superfamily level), which is attributable solely to the difference in the number of taxonomic units (*S* = 37 vs. *S* = 11), as the total specimen count (*N* = 8446) was identical across both scales. This corroborates previous assessments identifying Margalef richness as the index most responsive to variation in taxonomic unit counts [51]. Our findings underscore the necessity of explicitly reporting the taxonomic resolution employed when presenting richness indices and caution against direct inter-study comparisons of values derived at different taxonomic scales.

### 4.6. Implications for Conservation and Sustainable Pest Management

The functional structure documented in this study has direct implications for pest management in tropical pummelo orchards. Parasitoid dominance (73.30%) indicates that these orchards harbor substantial natural enemy diversity with potential for biocontrol, but this potential is vulnerable to insecticide-induced disruption. The use of biological pesticides (biopesticides) in the surveyed orchard, rather than broad-spectrum chemical insecticides, likely explains the persistence of this diverse hymenopteran community (191 morphospecies) under conventional management. This reduced-risk chemical regime should be considered when interpreting our results: the community described here reflects a management system that is already partially aligned with conservation biological control principles. We recommend three management strategies: (1) replace calendar-based spraying with threshold-based or hotspot-targeted application of broad-spectrum insecticides; (2) maintain flowering understory vegetation within orchards and adjacent hedgerows to support parasitoid and pollinator populations beyond the pummelo flowering season; (3) minimize insecticide application during peak hymenopteran activity periods to preserve predator and pollinator guilds [1,8,52]. These measures align with conservation biological control principles and may reduce chemical dependence while maintaining pest suppression efficacy.

From a conservation perspective, the 191 morphospecies documented across 37 families indicate that pummelo orchards can serve as refugia for hymenopteran biodiversity in tropical agricultural landscapes. However, this capacity is contingent on habitat management that preserves floral resources, nesting substrates, and alternative host populations. The historical shift from natural-enemy-rich citrus agroecosystems to chemically dependent systems [8] underscores the risk that intensive management poses to the parasitoid-dominated community documented here. Long-term monitoring of hymenopteran community responses to management interventions, such as organic conversion, intercropping, or reduced-spray regimes should employ replicated protocols that track both taxonomic and functional shifts. Although COI barcoding was employed here to confirm species-level identities for 24 morphologically ambiguous specimens (representing 16 morphospecies across 8 families), high-throughput metabarcoding offers complementary advantages for community-level surveys, particularly in detecting cryptic diversity and rare taxa that may elude traditional morphological methods [14,33,53]. Integrating metabarcoding with morphological identification would thus improve diversity estimates and strengthen the empirical basis for conservation-oriented management in tropical orchard systems.

## 5. Conclusions

This study provides the first morphospecies-level inventory of Hymenoptera in a low-spray, conventionally managed tropical pummelo orchard in China, documenting 8446 specimens belonging to 11 superfamilies, 37 families, and 191 morphospecies from Xishuangbanna. Although spatial replication was limited to a single orchard, the results demonstrate that pummelo orchards in this region harbor a morphospecies-rich and functionally diverse hymenopteran community that is overwhelmingly dominated by parasitoids (73.30%), with substantial contributions from predators (15.71%) and pollinators (9.42%), indicating a strong functional bias toward natural-enemy-mediated ecosystem services. Diversity analysis at two taxonomic resolutions demonstrated that family-level indices captured greater heterogeneity than superfamily-level estimates, underscoring the necessity of multi-scale assessments in orchard biodiversity surveys. This study provides a taxonomic and functional inventory of Hymenoptera in tropical pummelo orchards, demonstrating the importance of parasitoid-dominated communities for potential ecosystem service delivery in perennial tropical agroecosystems.

## Figures and Tables

**Table 1:** Morphospecies composition of Hymenoptera in pummelo orchards at the superfamily levels.

No.	Superfamily	Number of Morphospecies	Ratio
I	Ichneumonoidea	42	21.99%
II	Chalcidoidea	35	18.32%
III	Vespoidea	30	15.71%
IV	Apoidea	28	14.66%
V	Chrysidoidea	16	8.38%
VI	Cynipoidea	12	6.28%
VII	Platygastroidea	11	5.76%
VIII	Proctotrupoidea	9	4.71%
IX	Evanioidea	5	2.62%
X	Ceraphronoidea	2	1.05%
XI	Mymarommatoidea	1	0.52%

**Table 2:** Morphospecies composition of Hymenoptera in pummelo orchards at the family levels.

No.	Superfamily	Family	Number of Morphospecies	Ratio
I	Ichneumonoidea	Ichneumonidae	25	13.09%
		Braconidae	17	8.90%
II	Chalcidoidea	Eulophidae	9	4.71%
		Chalcididae	6	3.14%
		Encyrtidae	6	3.14%
		Mymaridae	3	1.57%
		Torymidae	3	1.57%
		Pteromalidae	2	1.05%
		Tanaostigmatidae	2	1.05%
		Aphelinidae	1	0.52%
		Ormyridae	1	0.52%
		Perilampidae	1	0.52%
		Tetracampidae	1	0.52%
III	Vespoidea	Pompilidae	11	5.76%
		Formicidae	9	4.71%
		Tiphiidae	6	3.14%
		Mutillidae	3	1.57%
		Scoliidae	1	0.52%
IV	Apoidea	Halictidae	9	4.71%
		Apidae	7	3.66%
		Crabronidae	6	3.14%
		Megachilidae	2	1.05%
		Pemphredonidae	2	1.05%
		Sphecidae	2	1.05%
V	Chrysidoidea	Bethylidae	13	6.81%
		Embolemidae	2	1.05%
		Chrysididae	1	0.52%
VI	Cynipoidea	Charipidae	4	2.09%
		Cynipidae	3	1.57%
		Eucoilidae	3	1.57%
		Figitidae	2	1.05%
VII	Platygastroidea	Scelionidae	11	5.76%
VIII	Proctotrupoidea	Diapriidae	7	3.66%
		Heloridae	2	1.05%
IX	Evanioidea	Evaniidae	5	2.62%
X	Ceraphronoidea	Ceraphronidae	2	1.05%
XI	Mymarommatoidea	Mymarommatidae	1	0.52%

Note: Percentages in this table are rounded to two decimal places. The cumulative rounding error (−0.02%) results in a total of 99.98%. This does not affect the consistency of morphospecies counts between Table 1 and this table.

## Data Availability

The data supporting the findings of this study are available within the article and its Supplementary Materials. Voucher specimens are deposited in Laboratory 329, Citrus Research Group, Yunnan Institute of Tropical Crops, Yunnan Province, China. DNA sequences generated during this study have been deposited in GenBank under accession numbers [PZ761219–PZ761242]. Additional data are available from the corresponding author upon reasonable request.

## Supplementary Materials

The following supporting information can be downloaded at https://www.mdpi.com/article/10.3390/insects17090886/s1, Figure S1: Superfamily-level community structure; Figure S2: Family-level community structure; Figure S3: Functional composition; Figure S4: Original photograph of a parasitoid wasp associated with citrus leaf miner (*Phyllocnistis citrella*) observed in the surveyed pummelo orchard; Figure S5: Representative photographs of Hymenoptera activity in the surveyed pummelo orchard; Table S1: Morphospecies composition at the superfamily level; Table S2: Morphospecies composition at the family level; Table S3: Voucher specimen data (complete inventory of the 191 hymenopteran morphospecies-level units recorded in the pummelo orchard); Table S4: Specimen abundance by family and collection method; Table S5: Functional composition of the hymenopteran community.
