# Supplementary material for: The Diversity of Hymenoptera on Pummelo (Citrus maxima (Burm.) Merr.)
Source: Insects. 2026 Aug 24;17(9):886. doi: 10.3390/insects17090886 (PMC13607962; doi:10.3390/insects17090886)
Supplement: Supplementary file 1 [file insects-17-00886-s001.zip › Table S1.pdf]

No.	Superfamily	Number of Morphospecies	Ratio
I	Ichneumonoidea	42	21.99%
II	Chalcidoidea	35	18.32%
III	Vespoidea	30	15.71%
IV	Apoidea	28	14.66%
V	Chrysidoidea	16	8.38%
VI	Cynipoidea	12	6.28%
VII	Platygastroidea	11	5.76%
VIII	Proctotrupoidea	9	4.71%
IX	Evanioidea	5	2.62%
X	Ceraphronoidea	2	1.05%
XI	Mymarommatoidea	1	0.52%
