# Supplementary material for: The Diversity of Hymenoptera on Pummelo (Citrus maxima (Burm.) Merr.)
Source: Insects. 2026 Aug 24;17(9):886. doi: 10.3390/insects17090886 (PMC13607962; doi:10.3390/insects17090886)
Supplement: Supplementary file 1 [file insects-17-00886-s001.zip › Table S2.pdf]

No.	Superfamily	Family	Number of Morphospecies	Ratio
I	Ichneumonoidea	Ichneumonidae	25	13.09%
		Braconidae	17	8.90%
II	Chalcidoidea	Eulophidae	9	4.71%
		Chalcididae	6	3.14%
		Encyrtidae	6	3.14%
		Mymaridae	3	1.57%
		Torymidae	3	1.57%
		Pteromalidae	2	1.05%
		Tanaostigmatidae	2	1.05%
		Aphelinidae	1	0.52%
		Ormyridae	1	0.52%
		Perilampidae	1	0.52%
		Tetracampidae	1	0.52%
III	Vespoidea	Pompilidae	11	5.76%
		Formicidae	9	4.71%
		Tiphiidae	6	3.14%
		Mutillidae	3	1.57%
		Scoliidae	1	0.52%
IV	Apoidea	Halictidae	9	4.71%
		Apidae	7	3.66%
		Crabronidae	6	3.14%
		Megachilidae	2	1.05%
		Pemphredonidae	2	1.05%
		Sphecidae	2	1.05%
V	Chrysidoidea	Bethylidae	13	6.81%
		Embolemidae	2	1.05%
		Chrysididae	1	0.52%
VI	Cynipoidea	Charipidae	4	2.09%
		Cynipidae	3	1.57%
		Eucoilidae	3	1.57%
		Figitidae	2	1.05%
VII	Platygastroidea	Scelionidae	11	5.76%
VIII	Proctotrupoidea	Diapriidae	7	3.66%
		Heloridae	2	1.05%
IX	Evanioidea	Evaniidae	5	2.62%
X	Ceraphronoidea	Ceraphronidae	2	1.05%
XI	Mymarommatoidea	Mymarommatidae	1	0.52%
